# Climate change impacts on optimal habitat of *Stachys inflata* medicinal plant in central Iran

**DOI:** 10.1038/s41598-023-33660-8

**Published:** 2023-04-21

**Authors:** Mehdi Shaban, Elham Ghehsareh Ardestani, Ataollah Ebrahimi, Massoud Borhani

**Affiliations:** 1 Department of Rangeland and Watershed Management, Faculty of Natural Resources and Earth Sciences, Shahrekord University, Shahrekord, 8818634141 Iran; 2 Central Laboratory, Shahrekord University, Shahrekord, 8818634141 Iran; 3 Natural Resources Research Division, Isfahan Agricultural and Natural Resources Research and Education Center, AREEO, Isfahan, Iran

**Keywords:** Ecology, Restoration ecology

## Abstract

*Stachys inflata* Benth. is a perennial shrub plant, with powerful natural antioxidant agents, which is recognized as a famous medicinal plant that is widely applied to treat Infection, Asthma, and Rheumatism. Iran is renowned as a center of diversity for Stachys, however, the ideal habitats of *S. inflata* in this nation remain unknown. The potential and future distribution of suitable habitats for *S. inflata* were projected using an ensembles ecological niche model in Isfahan province, Iran. We used occurrence data (using GPS), bioclimatic and topographic variables from the Chelsa and WorldClim databases to model the current and future potential distribution of this valuable species. The results showed that: (i) *S. inflata* is mainly distributed in the south, southwest, center, and west of the Isfahan province, and the excellent habitats of *S. inflata* accounted for 14.34% of the 107,000 km^2^ study area; (ii) mean annual temperature, mean daily temperature of wettest quarter, annual precipitation, and elevation were the four most important variables that affect the distribution of *S. inflata*, with a cumulative contribution of 56.55%; and (iii) about the half (− 42.36%) of the currently excellent habitats of *S. inflata* show a tendency to decrease from now to the 2080s, while often the area of other *S. inflata* habitats increases (the area of unsuitable habitat: 5.83%, the area of low habitat suitability: 24.68%, the area of moderate habitat suitability: 2.66%, and the area of high habitat suitability: 2.88%). The increase in the area of other *S. inflata* habitats is different and they are less favorable than the excellent habitat. The results help establishing a framework for long-term in-situ and ex-situ conservation and management practices in habitats of *S. inflata* in rangeland and agricultural ecosystems.

## Introduction

Iran is a vast country in Southwest Asia with many semi-arid to arid rangelands^1^. This country is located in the Irano-Turanian, the Saharo-Sindian and the Euro-Siberian regions (phytogeographic regions)^2^ and covers Irano-Anatolia and Caucasus, which are two global biodiversity hotspots^3,4^. With more than 8000 plant species, this country has high plant diversity, approximately 28% (2300 plant species) of which are aromatic and medicinal plants^5^.

The aromatic and medicinal plants are of great use to the Iranian people, not only for traditional treatment but also as a source of income, and they have the potential for greater worldwide use in the present and future^6^. We include these plants as a component of biodiversity and as a service to the environment^7^. Threats to medicinal plants in Iran can derive from deforestation, land-use change, overexploitation, climate change, rangeland and forest fires, herbivorous animals, invasive species, land and water pollution, mining industries, and different natural disasters^6^. Therefore, Iranian medicinal plants should be conserved and managed. Through the cultivation of medicinal plants, local communities may lessen their ecological footprint or impact on the natural environment and its resources. They also lead to prevent over-exploitation and extinction of this species by choosing the alternatives such as the cultivation of this plant, the adoption and adaptation of enclosures in rangelands, and biological-oriented soil and water conservation practices in rangelands.

Stachys is one of the greatest genera of Labiatae or Lamiaceae family (subfamily Laminoideae), includes over 275 species worldwide, and consists of herbaceous plants (annual or perennial herbs), sub-shrubs, and woody shrubs covered with various hairs^8,9^. The majority of species in this genus inhabit alpine and subalpine environments, as well as mountain steppe, stream banks, and woodlands. In general, Stachys grows in mountain regions with low temperature and rainfall indicated the high ecological flexibility of this genera. Climate change affects growth, physiological processes, and production of primary and secondary metabolites in this genera. In addition, the mechanism, behavior, or potential response of the Stachys are widely varied to climate factors^10^. Iran has 35 species of this genus, of which 13 are endemic, and is renowned as a diversity hub for Stachys^11^. This genus contains essential medicinal plants that are noted for their scent and utilized in traditional medicine. They are widely used in Asia and Europe as aromatic herbal teas. The essential oils made by these plants are employed in the drinks, food, drug, cosmetics, and perfume industries^9^.

*Stachys inflata* Benth. in the genus Stachys grows wild in some parts of the world such as Iran (Persian names: Ghole Arghavan, Poulk), Turkey, Iraq, Armenia, Azerbaijan, South Korea, the United States of America, Australia, and Switzerland^12^ (https://www.gbif.org), its native range is the Mediterranean and Irano-Turanian regions. This plant, with 20–40 (> 40) cm height, is a perennial shrub plant with red-pink flowers. This species is highly adapted to hot and dry to cold and dry mountainous regions. *S. inflata* is a powerful natural antioxidant agent (e.g., hexahydrofarnesyl acetone, germacrene D, α-pinene, β-pinene, and valeranone)^9^ and a recognized aromatic and famous medicinal plant which is widely applied to treat infection, asthma, rheumatism and known as an analgesic, anti-inflammatory and antiseptic^13–17^. Therefore, with the known properties of this species, it expanded the conservation and cultivation of plant resources obtained from this plant^9^. In previous studies on *S. inflata* different chemical compositions were reported over the recent decades^12,18,19^.

Medicinal plants are currently recognized as a source of medicine in many countries and are used in many cultures. In general, 30% of medicinal products are of plant origin. The distribution, variety, and quality of pharmaceutical compounds and the plant itself in medicinal plants are influenced by environmental conditions, particularly climatic variables on large scale^6^. Concerning climate change, the identification of climate variables’ effect on medicinal plant habitats is necessary for the conservation and management of medicinal plants like *S. inflata*.

Most of the earth experienced a warming trend and climate change over the last 30 years due to human activities which has widespread effects on the environment such as glaciers and ice sheets shrinking, plant and animal geographic ranges shifting, etc. In recent years, habitat loss in arid and semiarid areas increased by both natural drivers and human activity-driven climate change^8^. Across Iran, drought caused by climate change and human effects is impacting this country’s ecosystems, for instance, the drying of lakes, the disappearance or change of the range of habitats of some animal and plant species, etc. As an area in the desert belt, this nation is susceptible to the impacts of climate change. By rising temperatures in terms of climate change by 2100, it is predicted that many habitats of medicinal and aromatic plants in arid and semi-arid regions of this country will be lost^20^. To evaluate plant species' vulnerability under climate change, species distribution modeling constitutes the most widely used modeling framework in climate change impact assessments for estimating potential future range shifts of species^21^. Few studies have examined the effects of climate change on medicinal and aromatic plants^22^ such as *Aconitum spicatum*, *Allium wallichii*, *Bergenia ciliata*, *Nardostachys jatamansi*, *Neopicrorhiza scrophulariiflora*, *Paris polyphylla, Valeriana jatamansi*^23^*, Nepeta glomerulosa*^24^, *Salvia hydrangea*^25^, *Nepeta binaloudensis*^21^, *Fritillaria imperialis*^26^.

Researchers may use species distribution models to analyze the possible effects of climate change on species distribution, reveal the range of the species’ preferred habitat, and assess the potential effects of climate change on biodiversity^27–34^. To reduce uncertainties in the modeling, ensemble species distribution modeling is an approach where several different foundation models are employed to project an outcome^30,35–38^.

Climate change is affecting the medicinal plant distribution, the variety, quantity and quality of pharmaceutical compounds, and the way they interact with their habitats. To stop further habitat fragmentation and loss of medicinal plants, it is important to develop adequate conservation measures and strategies that it requires a comprehensive understanding of the relationship between the distribution range of species and climate change. Hence, investigating the relationship between the distribution range of species and climate variables to project the impact of climate change on the distribution range of species and offering conservation measures, has a very important significance for future medicinal plant conservation.

There is currently little research to determine the environmental factors affecting the conservation and cultivation of *S. inflata*^39^. Still, there is no knowledge about the response of this species to the effects of future climate change. Also, considering the useful properties of this plant, especially in therapeutic applications. Therefore, we predict the distribution of *S. inflata* under current and future conditions (in 2050 and 2080 based on three scenarios of increasing greenhouse gases (SSP126, SSP370, and SSP585), and as well as two general circulation models (GFDL-ESM4 and MRI-ESM2-0) in Isfahan province, Iran to prioritize conservation and management of this valuable plant species.

## Methods

### Study area

Isfahan province, with an area of 107,000 km^2^, is located in central Iran (31°26′–34°30′ N, 49°30′–55°50′ E), and it is bordered by Qom, Markazi, and Semnan provinces in the north, South-Khorasan and Yazd provinces in the east, Fars and Kohgilluyeh-va-Boyer Ahmad provinces in the south, and Chaharmahal-va-Bakhtiari and Lorestan provinces in the west (Fig. 2). The desert plain in the east and north, and the Zagros Mountains in the west and south, naturally confine the territory. For the province, this natural condition provides both advantages and disadvantages. The average altitude of Isfahan province is 1500 m above sea level (ranging from < 750 to > 4000 m) with total annual precipitation of 160 mm (38–1024 mm) and an average temperature of 19.5 °C (ranging from < 10 to > 22 °C). The climate of the study area varies from arid and semi-arid in the east and north to cold steppe and cold xeric in the south and west. Most precipitation in the study area occurs in the west and south during autumn, winter, and spring (October to May months). Over the past decades, low precipitation and high temperature have caused the extinction of plant species in many areas of Isfahan province^40^.

### Occurrence data collection

We collected 90 occurrence data (presence-only) for *S. inflata* throughout the Isfahan province from May to July (2020–2021) using GPS (global positioning system). Then, very knowledgeable experts about the distribution of the plant in the study area checked the data records^29^. We removed duplicate data, coordinate errors, and data-situated occurrence points closer than 1 km to each other^41^. We used 66 of 90 presence points of this species for modeling.

Pseudo-absence or background data are only generated in locations where there are no presence data and are equivalent to the number of presence data using randomly sampling grid cells across Isfahan province. We filtered background points by randomly selecting a background point within a single grid cell (1 × 1 km)^25^.

### Environmental variables

Many investigators have noted that more notice should be given to explanatory skills and ecological foundations to select environmental variables. Predicting a species' ecological niche requires determining the most critical environmental elements. Bioclimatic parameters and topography layers are used in ecological niche models to anticipate the distribution of species anywhere on the globe^42,43^. We chose three topographic and 19 bioclimatic layers (1981–2010) from WorldClim (http://www.worldclim.org) and the Chelsa (http://www.chelsa.org) databases both having a spatial resolution of 30 s (ca. 1 km)^44^. These bioclimatic layers were derived variables from the mean precipitation and monthly mean, max, and mean annual temperature values which provide information on temperature and precipitation globally at kilometer resolution to produce biologically significant layers and predicted species' ecological niches across large scales. These layers represent annual trends, seasonality, and extreme or limiting environmental variables. The CHELSA provided climate data as monthly and seasonal statistics averaged over a representative period of 30 years or longer^45^.

Projects for the future climate of Isfahan province were taken from the Coupled Model Intercomparison Project Phase 6 (CMIP6), as prepared by the IPPC in its sixth assessment report (AR6) ^46^. In this survey, we predicted future habitat suitability for *S. inflata* by the output of two GCMs: the first GCM named the GFDL-ESM4 (National Oceanic and Atmospheric Administration, Geophysical Fluid Dynamics Laboratory, Princeton, NJ 08,540, USA) and the second GCM named the MRI-ESM2-0 (Meteorological Research Institute, Tsukuba, Ibaraki 305–0052, Japan). The first has doubled the horizontal resolution of both atmosphere (2 to 1°) and ocean (1 to 0.5°) and the native resolution of 288 × 180 ^47^. The latest has horizontal resolution of 1.25° for longitude and latitude and the native resolution of 320 × 160^47,48^. We used these two GCMs because they showed appropriate temperature and precipitation forecasting when compared with the real data obtained from different synoptic stations in Iran. Thought, most GCMs (such as GFDL and MIR) perform reasonably appropriate in simulating the temperature and precipitation over Iran^49^. But, MRI-ESM2-0 model simulations are more consistent with the occurrence of wet and dry periods to the ground observations. Nonetheless, the wet and dry durations in GFDL-ESM4 model simulation are a little different from ground observations^47^. So, we estimated predictions of each of these models under three climate scenarios (SSP126, SSP370, and SSP585). We investigate future climate change conditions for the 2050s (average for years 2041–2070) and 2080s (average for years 2071–2100)^41,49,50^. The trend of temperature changes for the current (1981–2010) and the years 2050 and 2080 is 16.25 °C, 19.01 °C, and 19.96 °C respectively, and the trend of precipitation changes for this period is 218.40 mm, 224.74 mm, and 221.17 mm, respectively.

### Modeling

First, we employed Pearson's correlation coefficients to remove highly collinear environmental layers^30,51–53^. When two layers were found to be highly collinear (*|r|*> 0.8), we used only one layer for modeling.

**Figure 1:**
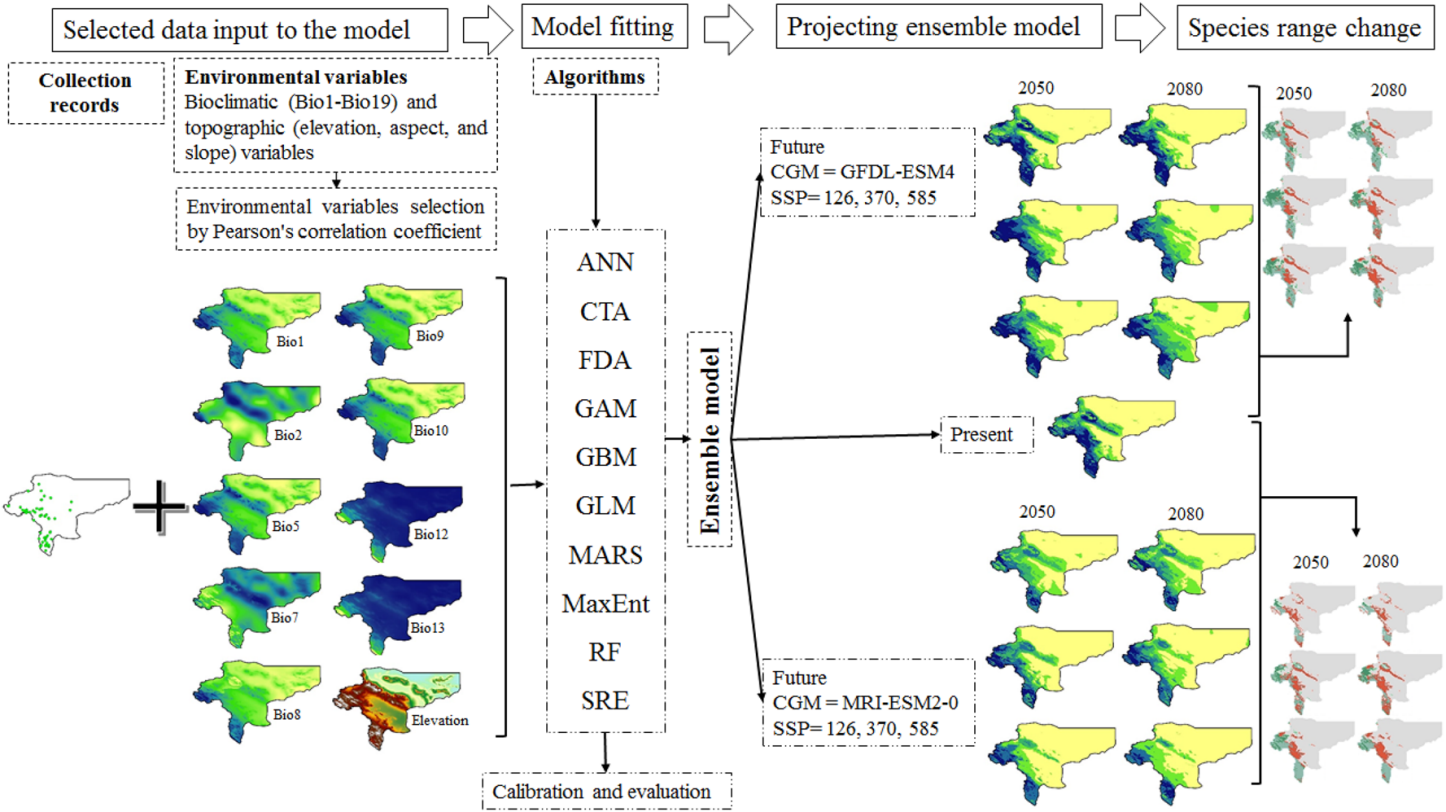
The modeling process of projected suitable habitat for *S. inflata* under current and future climate change conditions.

We applied the BIOMOD2 package in R to expand our ensemble ecological niche models for the geographic distribution of *S. inflata* in Isfahan province. We employed ten ecological niche models in Fig. 1:^54^ A cross-validation approach (a repeated data-splitting procedure) is carried out in our study because there is no independent dataset available to assess the models. We used 80% of the data as training data (calibration) and 20% of the data as test data (validation) randomly. This procedure is repeated ten times to calculate each model. Model accuracy was measured by AUC (Area under the receiver operating curve) and TSS (true skill statistic). AUC values were categorized into five groups: (1) invalid (≤ 0.6), (2) bad (0.6–0.7), (3) acceptable (0.7–0.8), (4) good (0.8–0.9), and (5) excellent (> 0.9). The models were ranked according to the TSS values to the 5 groups: 1) lacking predictive capability (≤ 0.2), 2) bad (0.2–0.4), 3) acceptable (0.4–0.6), 4) good (0.6–0.8), and 5) excellent (> 0.8)^54^.

The ensemble distribution model was derived from a weighted average of ten separate algorithms using the TSS evaluation scores (TSS > 0.8) that only models with a TSS greater than or equal to 0.8 are kept to build the final ensemble. The species occurrence probabilities (0–1) created using the ensemble ecological niche model were classified into five groups: (1) excellent habitat suitability (*p* ≥ 0.8), 2) high habitat suitability (0.6 ≤ *p* < 0.8), (3) moderate habitat suitability (0.4 ≤ *p* < 0.6), (4) low habitat suitability (0.2 ≤ *p* < 0.4), and (5) unsuitable habitat (*p* < 0.2). Then, the continuous habitat suitability predictions generated by the ensemble ecological niche model were converted into binary suitability maps (1 = suitable, 0 = not suitable). The thresholds are chosen based on the suitability value that maximizes a given metric that the threshold of maximization TSS (MAX_TSS threshold) was used the suitability value that gives the highest TSS value to create binary maps to convert the occurrence probability values into presence/absence predictions. This is the common threshold at which the sum of Specificity and Sensitivity is maximum^21,55,56^. We generated the binary suitability maps based on the MAX_TSS threshold using the “binary.meth” method from the BIOMOD package in R^55^.

## Results

First, we selected 22 environmental factors from the Chelsa and WorldClim datasets. Here, we use Pearson's correlation coefficients to visualize the correlation between the factors to identify the main factors in the region to enter the modeling process^55^.

Based on Pearson's correlation coefficients, ten environmental layers (seven Bioclim variables for temperature and two for precipitation, one for topography) were preferred for modeling (Table 1). Then, We run the models with all ten factors to see what their contributions are (Table 2).

**Table 1:** Environmental variables were employed to model *S. inflata* distribution pattern in the two climatic times (2050 and 2080).

Number	Short name	Long name	Unite	database
1	Bio1	Mean annual temperature	°C	Chelsa
2	Bio2	Mean diurnal temperature range	°C	Chelsa
3	Bio5	Mean daily maximum temperature of the warmest month	°C	Chelsa
4	Bio7	Annual range of temperature	°C	Chelsa
5	Bio8	Mean daily temperature of the wettest quarter	°C	Chelsa
6	Bio9	Mean daily temperature of the driest quarter	°C	Chelsa
7	Bio10	Mean daily temperature of the warmest quarter,	°C	Chelsa
8	Bio12	Annual precipitation amount	mm	Chelsa
9	Bio13	Precipitation amount of the wettest month	mm	Chelsa
10		Elevation	m	WorldClim

**Table 2:** Importance (%) of the environmental layers in *S. inflata* distribution algorithms. The bold variables show the two maximum relative contributions to each model in predicting suitable habitats for this species. The variable relative importance score showed mean variable relative importance in all ten algorithms. The contribution of each environmental variable is the relative percentage of that variable's relative importance compared to others variables. See other abbreviations as in Table 1 and Fig. 3.

Variable	Bio1	Bio8	Bio12	Elevation	Bio13	Bio10	Bio5	Bio9	Bio2	Bio7
GLM	22.72	45.29	17.90	8.56	8.02	**38.52**	5.15	1.26	5.24	0.36
GBM	6.19	24.60	8.76	1.87	2.13	0.04	0.55	0.81	3.01	1.76
RF	3.01	7.04	2.23	1.53	2.12	0.49	0.62	0.61	1.20	1.37
SRE	33.98	25.70	29.26	33.40	28.71	28.90	**27.97**	**29.20**	11.07	**12.25**
CTA	**79.42**	21.11	3.19	4.02	4.15	3.05	3.84	3.04	4.20	0.35
ANN	12.24	31.66	**81.98**	**44.31**	**46.18**	10.18	17.58	9.59	11.43	10.62
FDA	40.14	45.02	39.30	30.64	17.20	20.05	25.40	13.32	14.00	9.66
MARS	57.42	**52.60**	41.48	22.85	18.07	14.53	26.48	22.61	10.15	7.76
GAM	**65.44**	**52.20**	**74.51**	**65.15**	**71.66**	**74.54**	**64.98**	**72.32**	**43.18**	**50.07**
MAXENT	41.10	39.06	37.00	7.08	14.4	0.10	8.52	6.28	**16.47**	9.90
Relative importance	36.17	34.43	33.56	21.94	21.26	19.04	18.11	15.90	12.00	10.41
Contribution	16.22	15.44	15.05	9.84	9.54	8.54	8.12	7.13	5.38	4.67

### Model performance and contribution of environmental variables

**Figure 2:**
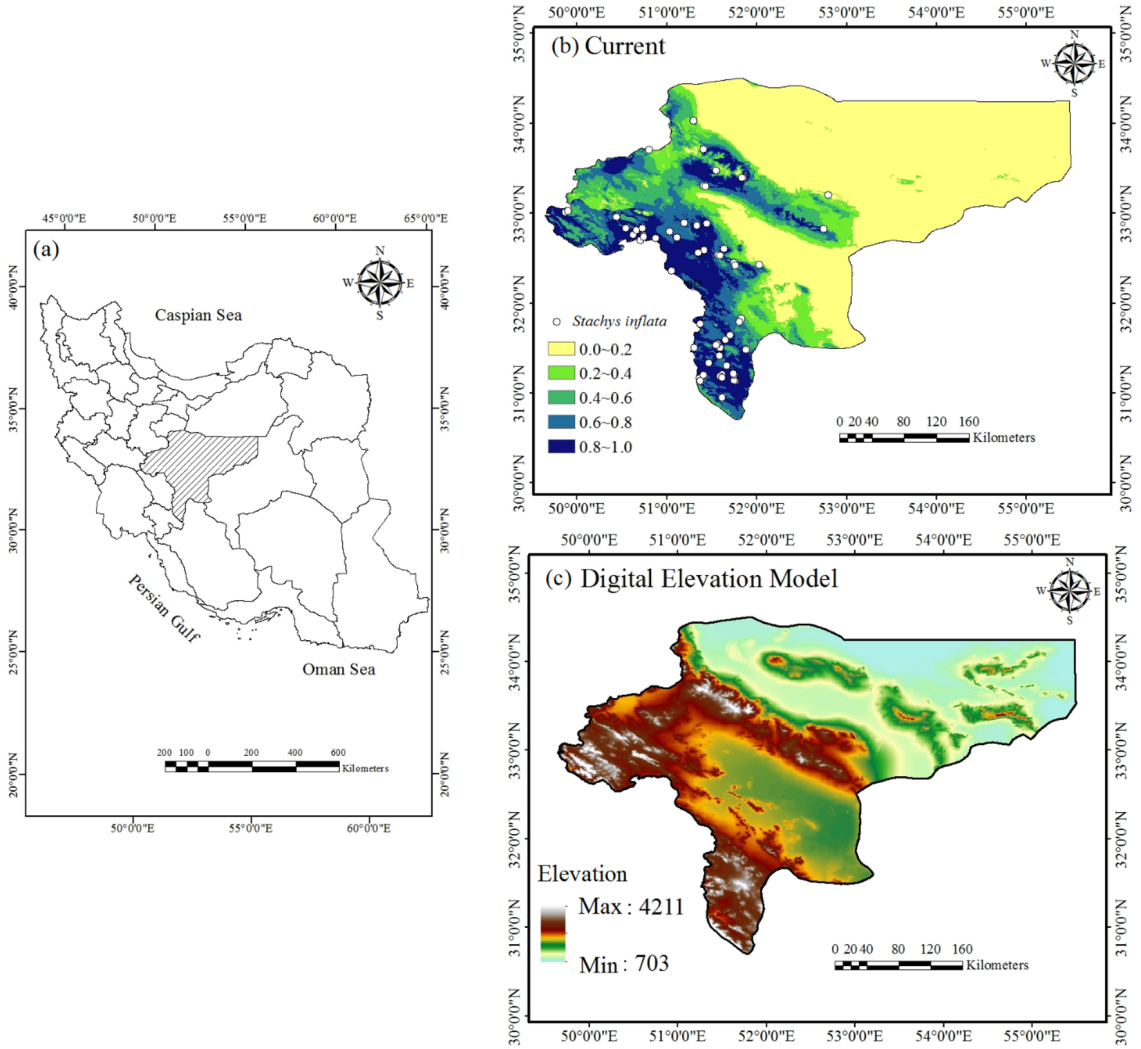
Isfahan Province is located in central Iran (**a**), the ensemble projects for climates presently suitable for *S. inflata* (**b**), and the digital elevation model (DEM) of Isfahan province (**c**).

The ensemble predictions revealed good qualitative compliance with the present geographical distribution of *S. inflata* in Isfahan province (Fig. 2).

**Figure 3:**
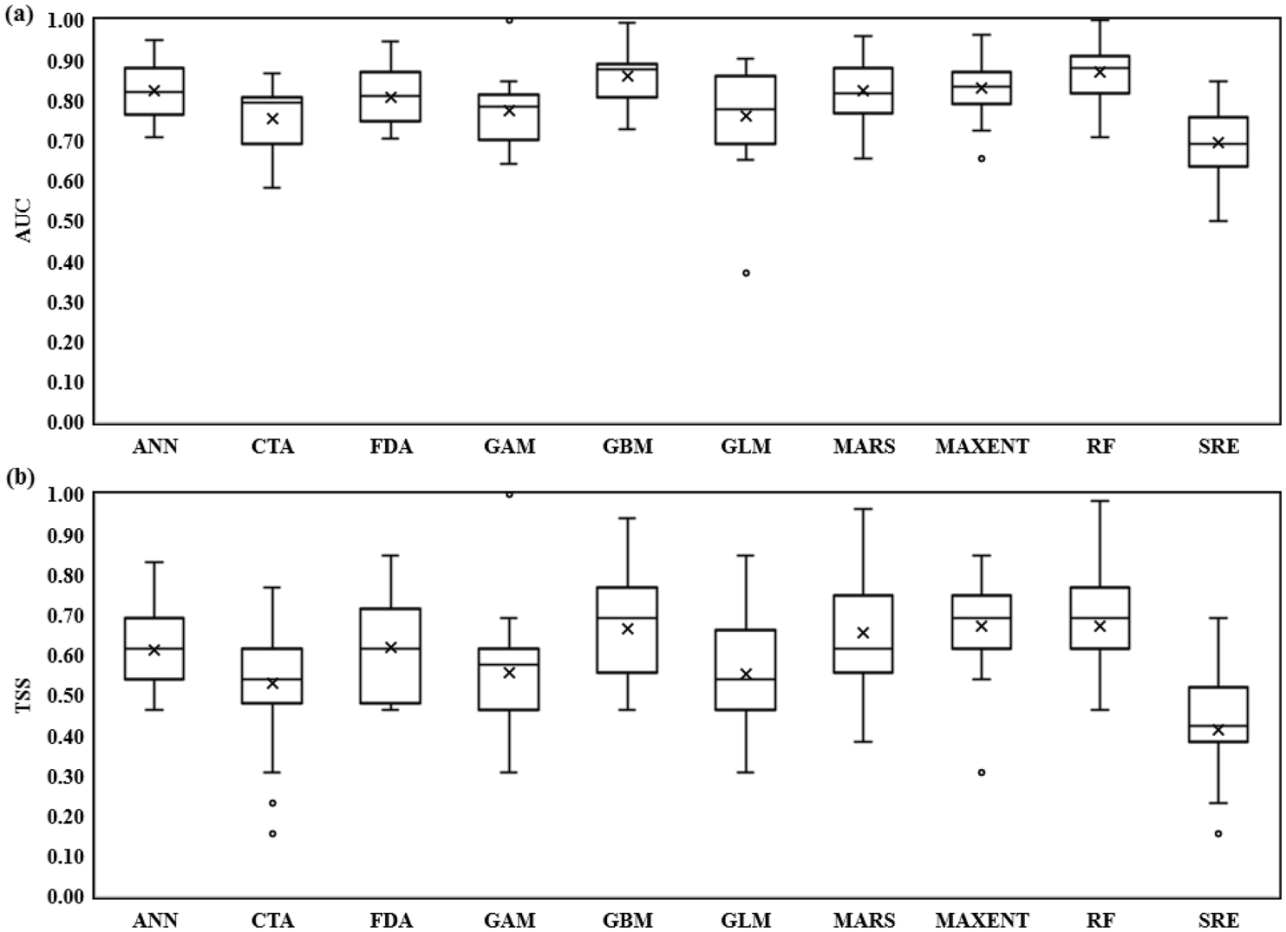
The indices of the AUC (Area Under Curve: (**a**), and the TSS (True Skill Statistic: (**b**) of ten modeling methods (ANN: Artificial Neural Network, CTA: Classification Tree Analysis, FDA: Flexible Discriminate Analysis, GAM: Generalized Additive Model, GBM: Generalized Boosted Model, GLM: Generalized Linear Model, MARS: Multivariate Adaptive Regression Splines, MaxEnt: Maximum Entropy, RF: Random Forest, and SRE: Surface Range Envelope) for predicting *S. inflata* distribution during the current periods.

Algorithms had acceptable to good performance to predict habitat distribution of *S. inflate* (AUC = 0.696–0.870, TSS = 0.413–0.673). The highest-performing algorithms were MaxEnt (TSS = 0.673) and RF (TSS = 0.672) for TSS. The lowest-performing algorithms were SRE (TSS = 0.413) and CTA (TSS = 0.530) (Fig. 3)^57^.

The variable relative importance score showed mean variable relative importance in all ten algorithms. The contribution of each environmental variable is the relative percentage of that variable's relative importance compared to others variables. The variable contribution scores showed the environmental variables that were influential in distinguishing the distributions of suitable habitats for *S. inflata*. The findings indicated that the selected layers accurately represented the current distribution of this species (Table 2). Among the ten chosen layers, mean annual temperature (Bio1), mean daily temperature of wettest quarter (Bio8), annual precipitation (Bio12), and elevation had the maximum contributions to model projects, and these layers together estimated about 56.55% of the total predictive power (Table 2).

**Figure 4:**
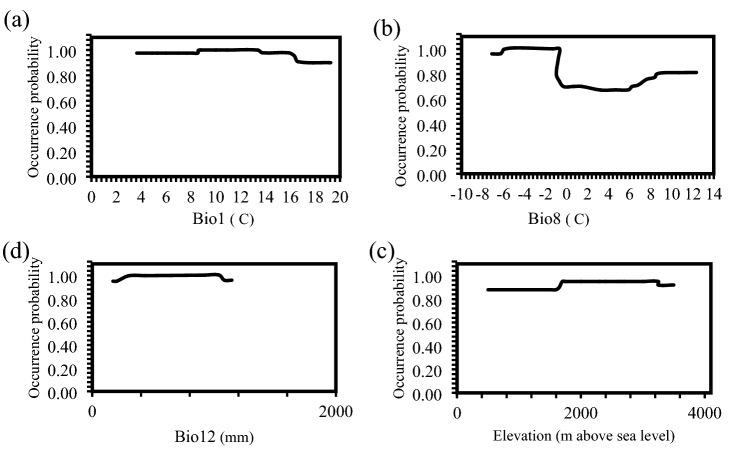
Response curves for the three Bioclimatic (**a**–**c**) and one topographic (**d**) layers used in the RF algorithm for *S. inflata.* See abbreviations as in Table 1.

Response curves created from the maximum performing algorithm that the highest performing algorithms had RF based on two evaluation criteria (AUC = 0.870; TSS = 0.672)^58^. Each curve is generated using the “response.plot” method from the BIOMOD package in R^59^. The RF algorithm, revealed that the area of excellent habitat suitability (*p* ≥ 0.8) for *S. inflata* located in regions with mean annual temperature (Bio1) from 3.5 to 19.7 °C, mean daily temperature of wettest quarter (Bio8) from − 8 to − 1°C, annual precipitation (Bio12) from 200 to 1200 mm and elevation from 500 to 3500 m above sea level (Fig. 4).

### Predicted current habitat distribution

To evaluate the area of suitable habitat for *S. inflata* in the present conditions, the area within the five habitat suitability classes was measured in Isfahan province (Fig. 2). The maximum regions of high to excellent habitat suitability (*p* ≥ *0.6*) were found in the south, southwest, west, and central Isfahan province (Fig. 2). According to Fig. 2, 14.34% of the 107,000 km^2^ study area was indicated as excellent suitability, 7.97% as high suitability, 8.45% as moderate suitability, 8.63% as low suitability, and 60.61% as unsuitable habitat for *S. inflata* under current conditions.

### Predicting future habitat distribution

**Figure 5:**
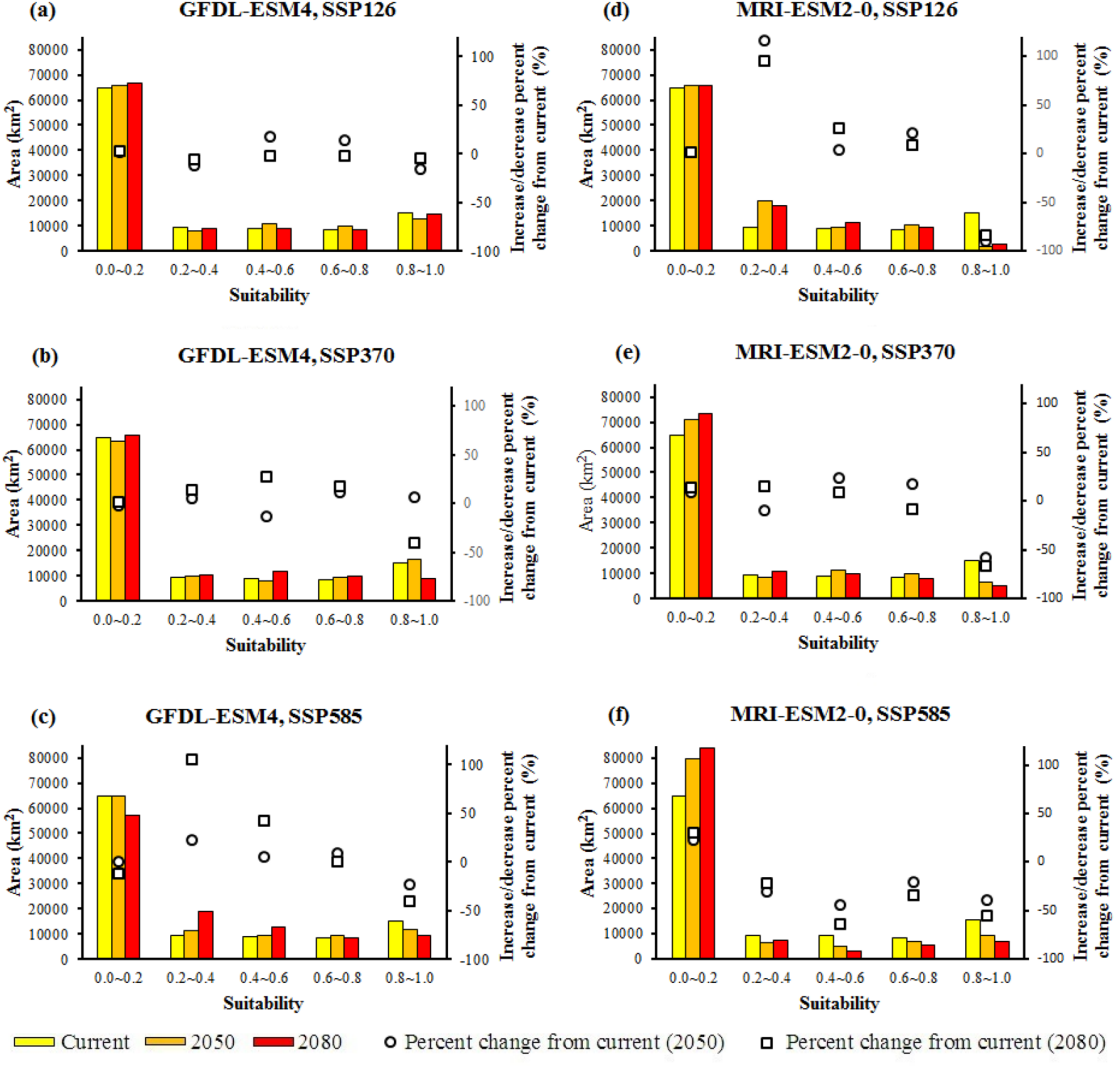
Projected suitable habitat for *S. inflata* under current and future climate change conditions (The percent change from current shows the proportion change from the current area in each suitability group). Each part refers to a different GCM (the first GCM: **a**–**c**; the second GCM: **d**–**f**) and different emissions scenarios.

Using the ensemble models, the future distribution of suitable habitats for *S. inflata* in Isfahan province was predicted using the two GCMs (the first GCM: GFDL-ESM4 and the second GCM: MRI-ESM2-0) under three climate change scenarios (the first scenario: SSP126, and the second scenario: SSP370, and the third scenario: SSP585) (Figs. S1 and S2; Fig. 5). The results showed significant differences among the current habitats and those projected for 2050 and 2080 under three scenarios.

**Figure 6:**
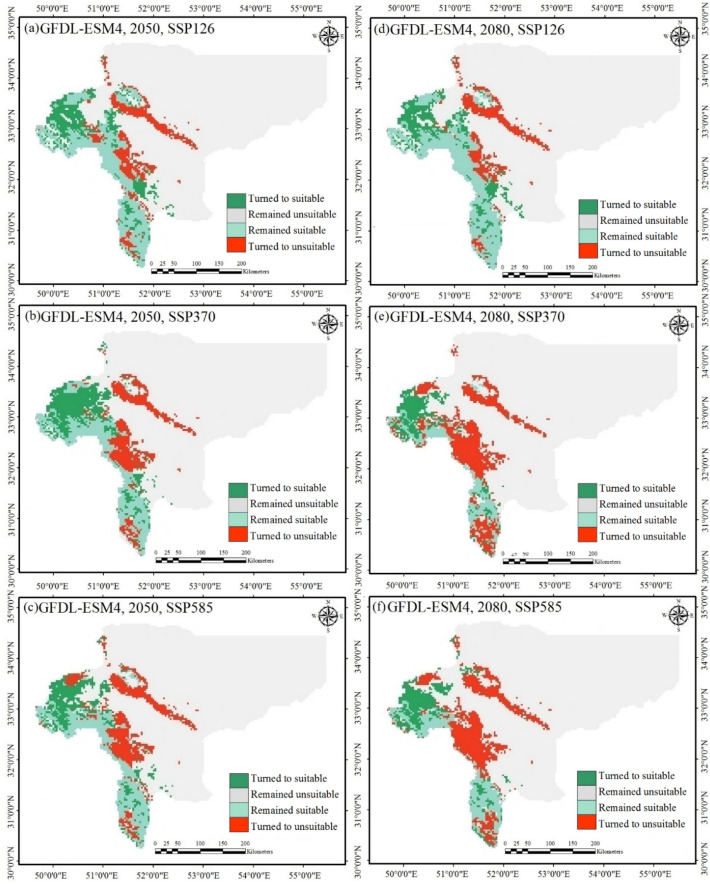
Changes in the suitable habitat distribution for *S. inflata* under current and the climates of 2050 (**a**–**c**) and 2080 (**d**–**f**) under the first GCM. Each part displays a different year and emissions scenario.

The area of excellent habitat suitability (*p* ≥ 0.8) would considerably shrink by − 4.03% to − 89.38% for both GCMs compared to the current distribution. This area would increase for the first GCM under the second scenario for 2050 (6.94%) compared to the current distribution condition (Figs. S1 and S2; Fig. 5).

Based on the climate prediction of the first GCM compared to the current distribution, the area of high habitat suitability (0.6 ≤ *p* < 0.8) would increase from 0.58 to 17.90%, and this area would shrink in the case of the first scenario for 2080 (− 2.62%) (Fig. S1; Fig. 5a–c). Under the climate projections of the second GCM, the area of high habitat suitability would increase from 9.17 to 20.79% under the first scenario (2050 and 2080) and the second scenario (2050) compared to the current distribution while this area would decrease from − 8.06 to − 34.28% under the second scenario (2080) and the third scenario (2050 and 2080) (Fig. S2; Fig. 5d–f).

Based on the climate predictions of two GCMs, the area of moderate habitat suitability (0.4 ≤ *p* < 0.6) would increase from 3.78 to 42.43%, except in the case of the first GCM under the first scenario for 2080 (− 2.20%) and the second scenario for 2050 (− 13.80%) and the second GCM under the third scenario for 2050 and 2080 (− 44.70, − 64.47%) (Figs. S1 and S2; Fig. 5).

Based on the climate prediction of the first GCM, the area of moderate habitat suitability (0.2 ≤ *p* < 0.4) would increase between 5.91 and 105.24%, this area would shrink in the case of the first scenario for 2050 and 2080 (− 12.36, − 5.66%) (Fig. S1; Fig. 5a–c). The projections based on climate predicted by the second GCM revealed a 15.02% to 116.24% increase in the area of moderate habitat suitability under the first scenario (2050 and 2080) and the second scenario (2080) while this area would decrease between − 8.91% and − 30.21% under the second scenario (2050) and the third scenario (2050 and 2080) (Fig. S2; Fig. 5d–f). Model predictions based on climate projected by the two GCMs generally showed an increase between 0.16 and 29.92% in the total area of the unsuitable habitat (*p* < 0.2), this area would shrink in the case of the first GCM under the second scenario for 2050 and the third scenario for 2080 (2.05–11.53% decrease in *p* < 0.2) (Figs. S1 and S2; Fig. 5).

**Figure 7:**
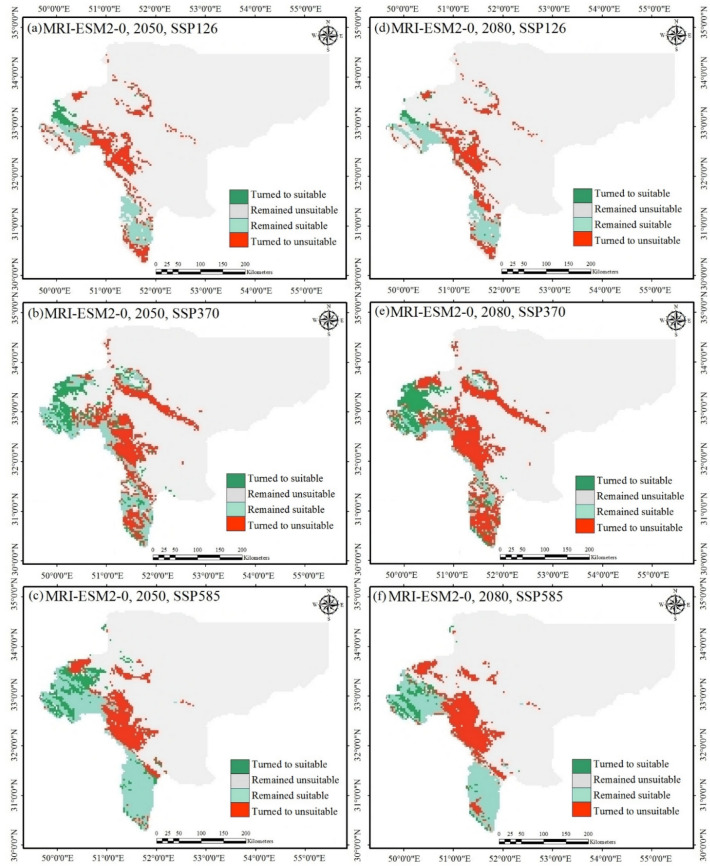
Changes in the suitable habitat distribution for *S. inflata* under current and the climates of 2050 (**a**–**c**) and 2080 (**d**–**f**) under the second GCM. Each part displays a different year and emissions scenario.

In summary, the ensemble ecological niche model suitability projections were categorized into binary suitable/unsuitable groups, and from these determined four categories of suitable habitat change: (i) stable presence is in the areas currently occupied by *S. inflata* and projected to stay occupied into the future (suitable to suitable), (ii) habitat loss are the areas that are currently suitable nevertheless projected to turn unsuitable in the future (suitable to unsuitable), (iii) habitat gain are areas that are currently not suitable nevertheless turn suitable in the future (unsuitable to suitable), (iv) stable absence are areas that are unsuitable in the current climate and stay so (unsuitable to unsuitable)^60^. Table 3 shows the areas falling into each of these four classes under three emissions scenarios for the years 2050 and 2080. Predictions for each category for two GCMs are shown in Figs. 6 and 7.

**Table 3:** Changes in the suitable habitat area for *S. inflata* under climate change conditions in Isfahan province compared to the current distribution in percent and absolute area changes.

GCM	Scenario	Year	Stable absence (km^2^)	Stable presence (km^2^)	Habitat loss (%)	Habitat gain (%)	Net change (%)
First:GFDL-ESM4	First:SSP126	2050	111,869	18,093	34.312	34.458	0.145
		2080	112,054	19,217	30.232	33.786	3.554
	Second:SSP370	2050	111,853	14,621	41.502	48.24	6.738
		2080	117,198	8505	65.972	26.854	− 39.117
	Third:SSP585	2050	112,867	13,943	47.329	36.133	− 11.197
		2080	114,267	10,053	62.024	30.844	− 31.180
Second: MRI-ESM2-0	First:SSP126	2050	130,689	6866	59.132	7.777	− 57.355
		2080	131,155	7594	54.687	5.907	− 48.780
	Second: SSP370	2050	116,164	11,850	55.273	23.575	− 31.698
		2080	115,600	6,655	74.881	25.704	− 49.177
	Third:SSP585	2050	115,958	16,730	38.979	20.166	− 18.813
		2080	118,306	13,903	49.291	11.602	− 37.688

The ensemble ecological niche model projected by 2050 that the area of habitat gain would total to 7.77% (the first scenario, the second GCM) to 48.24% (the second scenario, the first GCM) of the current geographic distribution, although the area of habitat loss would total to 34.31% (the first scenario, and the first GCM) to 59.13% (the first scenario, and the second GCM) of the current geographic distribution (Table 3; Figs. 6 and 7). Furthermore, the area of habitat gain of *S. inflata* would increase from 5.91 (the first scenario, and the second GCM) to 33.79% (the first scenario, and the first GCM) in 2080, yet would decrease from 30.23% (the first scenario, and the first GCM) to 74.88% (the second scenario, and the second GCM)in the same time (Table 3; Figs. 6 and 7). In four out of six model predictions for the year 2050, and five out of six model predictions for the year 2080, the ensemble ecological niche model predicted a net decrease in the area of suitable habitat for *S. infalata* (Table 3). The three exceptions to it, are the first GCM projections for the first scenario in 2050 and 2080, and the second scenario in 2050, which revealed a minor net increase in the area of suitable habitat (Table 3). Finally, from east to west of Isfahan province, the habitat suitable for *S. inflata* would decrease in the future (the first GCM projections under the second scenario for 2080, the first GCM projections under the third scenario for 2050 and 2080, the second GCM projections under three scenarios for 2050 and 2080).

## Discussion

Currently, excellent habitats of *S. inflata* are predicted in the west and south of Isfahan province. These medicinal plant habitats could progressively become compressed under future climate change. Climate change could induce upward shifting of this medicinal plant from the middle to high altitudes in Isfahan province. Therefore, The development of computer programs and approaches of species distribution models, especially the ensemble model, have enabled the conservation and management prioritization of medicinal plants under present climate conditions and the formulation of conservation plans to address the impacts of future climate change on these medicinal plants’ habitats^61–63^.

Based on the scores of the AUC and TSS indices, the performance of all ten algorithms was valid in the current climate conditions. The RF and SRE algorithms had the greatest and lowest performance, respectively, based on the scores of these indices^6^.

In general, the areas in the south and west are probably the most suitable habitat for *S. inflata* in Isfahan province. Moreover, the south and west of the study area are mountainous areas that contain more rainfall and higher average altitude, and lower average temperature, and these areas tend to have higher plant diversity.

The entire predicted suitable habitat under the current climate encompasses a larger region than the existence of this species discovered based on actual experimental results and verified by field surveys^40^. The current area of *S. inflata* could be increased as the results suggest a larger area suitable for its growth but barriers other than climate may limit the extent to which this plant spread. The barriers are major geographical barriers, environmental barriers (abiotic and biotic), and regular reproduction barriers such as physical barriers, nutrient or food availability, soil type, the presence of adequate breeding sites, land-use change, overexploitation, different natural disasters, etc.^64^.

This species distribution on the anticipated distribution map under the present climate was affected by various environmental factors. The most important environmental variables affecting the structure and function of plants are rainfall and temperature. In this study, annual mean temperature (Bio1, °C), mean daily temperature of wettest quarter (Bio8, °C), annual precipitation (Bio12, mm), and elevation (m above sea level) were the four most important bioclimatic factors in the creation of the species distribution models of *S. inflata*, and the accumulated their contributions exceeded 46.70%.

The highest suitability (*p* ≥ 0.8) of *S. inflata* occurred when mean annual temperature (Bio1) ranged from about 3.5 and 19.7 °C, mean daily temperature of wettest quarter (Bio8) ranged from about − 8 to − 1 °C, annual precipitation (Bio12) ranged from about 200 to 1200 mm, and elevation ranged from about 500 and 3500 m above sea level. Our results agree with the conclusion of Shahbazi et al.^65^, that *S. inflata* mainly grows in mountain regions with cold-dry climates^65^. This concurs with the preceding research showing *S. inflata* is a plant acclimated to the environmental factors of around 2200 m height, 275 mm annual precipitation, and 11.5 °C annual mean temperature. Elevation had a noticeable impact on *S. inflata* within our research^39,66^. Similar results were obtained by Gahyaningsih et al.^6^ and Ghehsareh Ardestani and Heidari Ghahfarrokhi^25^ in their studies on medicinal plant distribution found the same result^6,25^.

We find that *S. inflata* distribution was likely towards higher elevations (to shift upwards) with consistent warming across Isfahan province that both low temperature and water availability limit upward shifts at upper elevation limits. While we highlight the important topographic and bioclimatic variables limiting the distribution of *S. inflata*, more research is needed to understand how seed dormancy, germination, seed dispersal, pollination, and soil characteristics impact the survival of this species. We may need to carefully consider local variations in soil texture when planting *S. infata*. Better regeneration of this species appears to occur in sandy, silty, and clay-loam soils. Researches show that seed dormancy and insufficient soil moisture are the main limiting variables for the germination of this species in natural populations. In order to break seed dormancy, this plant must spend the cold winter in moist soils that this restriction is removed in mountain regions with cold-dry climates^66^.

Most predictions showed that the distribution areas of *S. inflata* in Isfahan would decrease in the future. One of the reasons, temperature increase might be expected to lead to suitable habitat loss for this species. Based on the reduction of suitable habitats for *S. inflata*, we introduced two approaches to identify habitats for conservation priorities. Where these projected suitable habitats overlap with currently suitable habitats (stable presence) of *S. inflata*, we suggest (i) in-situ conservation in these areas with “low threat level” due to climate change^67–69^. In these habitats, conservation along with sustainable exploitation is suggested (long-period in-situ conservation of this plant)^6^. Currently suitable habitats may become unsuitable habitats (habitat loss) for this *S. inflata* in the future, we propose (ii) ex-situ conservation in these areas with a “severe threat level” due to climate change. There are the habitats which will have the highest loss of this species in the future. Therefore, these habitats have a higher priority for conservation and management actions for sustainable exploitation in the future (ex-situ conservation of this plant)^67^. It is here assumed that *S. inflata* may lose presence in one habitat; nevertheless, this species has a chance to move to a habitat outside its favorable environmental factors and thrive at different altitudes or latitudes (Habitat gain) due to response to future climate change by dispersing its seeds (upward shifting habitats)^70,71^.

We examined only bioclimatic and topography variables. Future research could examine factors such as the interaction between individuals of this species and different species, and human activities (e.g., land-use change)^72^.

## Conclusion

We have predicted the distribution of *S. inflata* under current and future conditions in Isfahan province. This medicinal plant will upward shift to the west and south parts of this region. Climate change could induce the shifting of this medicinal species from the middle to high altitudes in this region. This study shows that excellent habitats of *S. inflata* are projected to be negatively affected because of future climate change. Therefore, *S. inflata* will be threatened in the future. Here, we have proposed two approaches for proper conservation (in and ex-situ conservation) of this species. Hence, the results of this study can be used to offer conservation measures and strategies for this valuable medicinal plant in the studied region.

## Supplementary Information

Supplementary Information.

Supplementary Figures.

## Data Availability

The datasets used and/or analysed during the current study available from the corresponding author on reasonable request.
