# Supplementary material for: Climate change impacts on optimal habitat of Stachys inflata medicinal plant in central Iran
Source: Sci Rep. 2023 Apr 21;13:6580. doi: 10.1038/s41598-023-33660-8 (PMC10121668; doi:10.1038/s41598-023-33660-8)
Supplement: Supplementary file 2 — Supplementary Figures. [file 41598_2023_33660_MOESM2_ESM.docx]

**Supplementary Information**


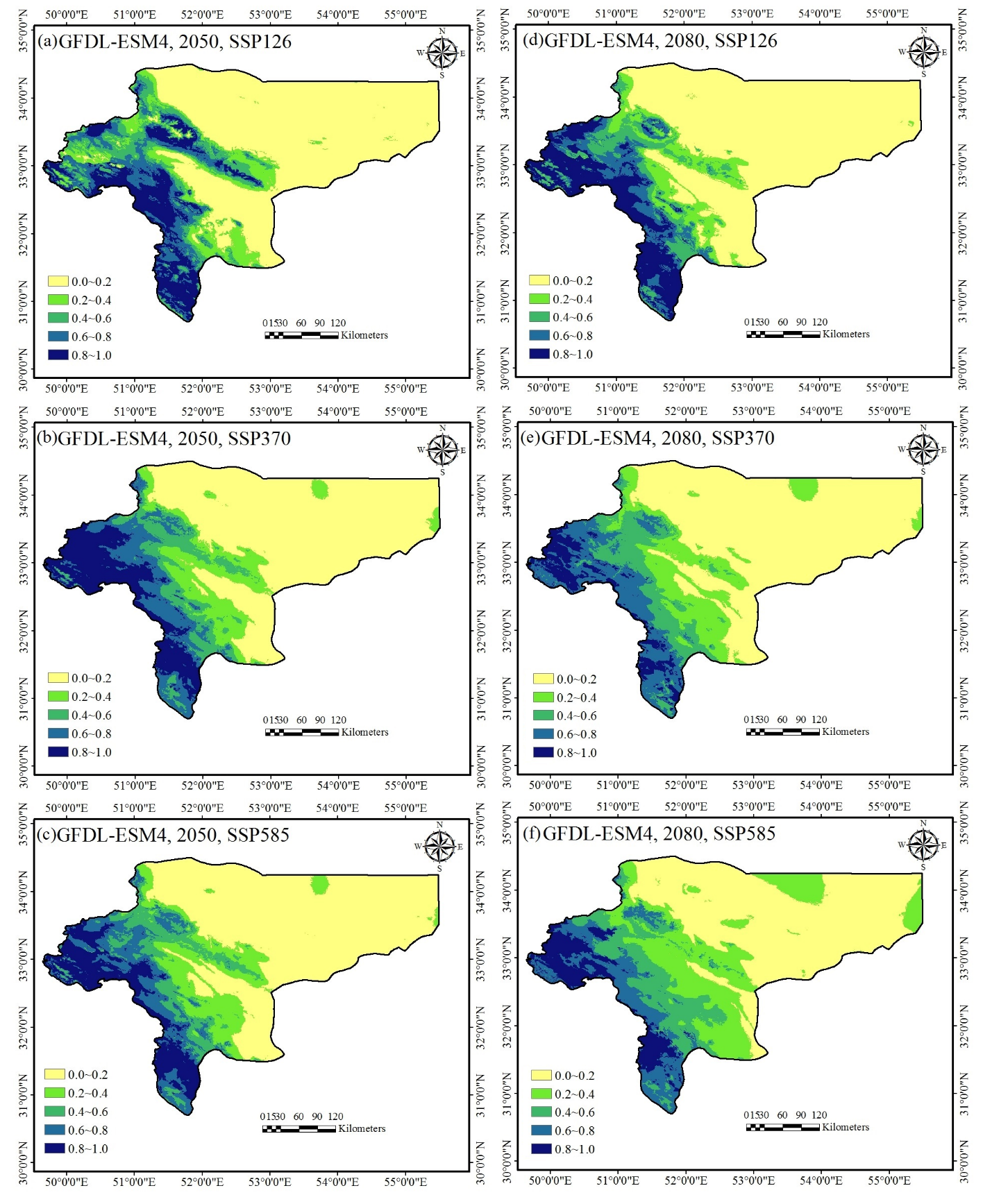


**Figure S.1.** Potential suitable distribution of *S. inflata* under future climate change predicted by the GFDL-ESM4 model. Each panel shows a different year (2050: a, b, c; 2080: d, e, f) and emissions scenario (SSP126, SSP370, SSP585).


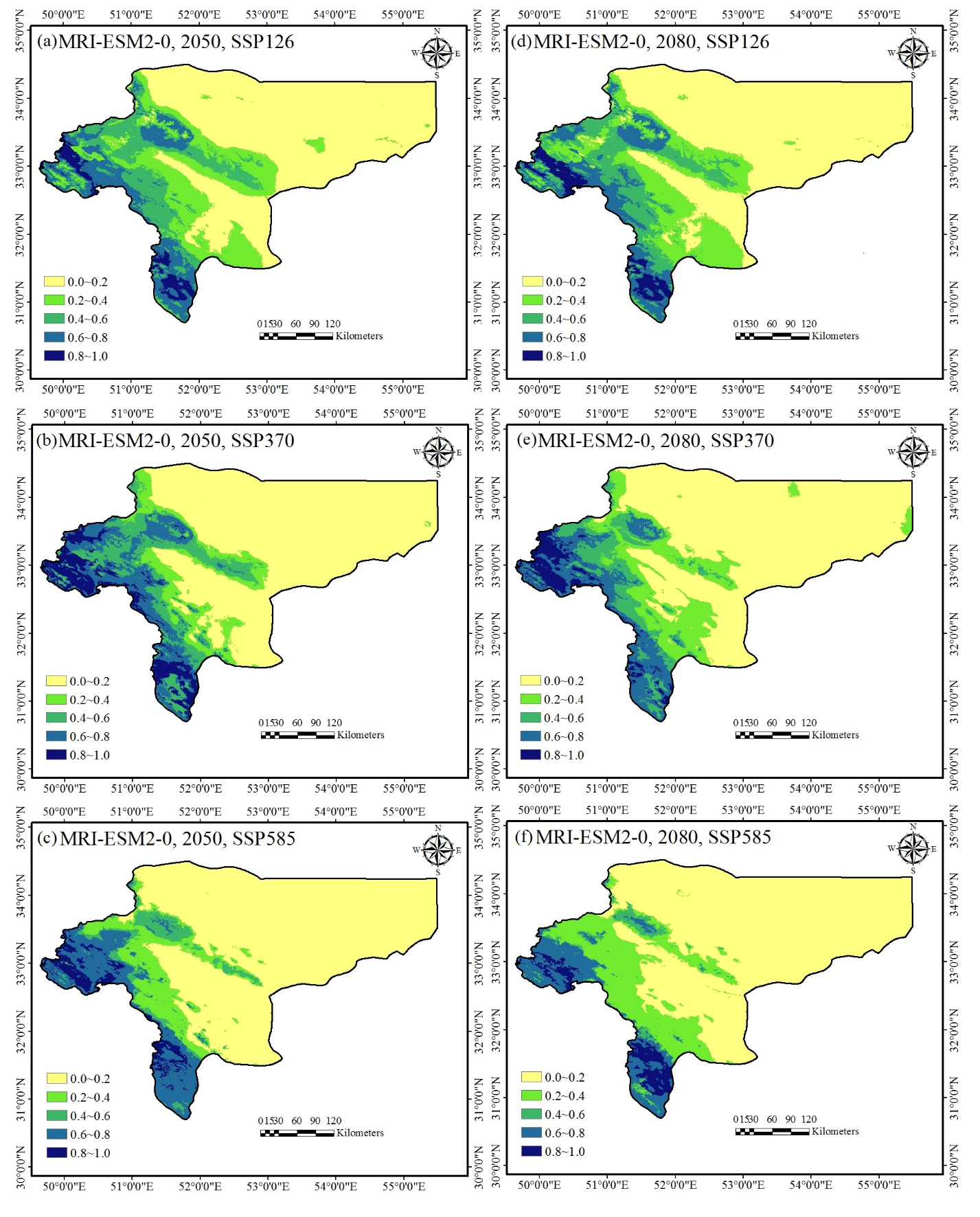
 **Figure S.2.** Potential suitable distribution of *S. inflata* under future climate change predicted by the MRI-ESM2-0 model. Each panel shows a different year (2050: a, b, c; 2080: d, e, f) and emissions scenario (SSP126, SSP370, SSP585).
